# Towards Enhanced Gas Sensor Performance with Fluoropolymer Membranes

**DOI:** 10.3390/s16101605

**Published:** 2016-09-28

**Authors:** Thorsten Graunke, Katrin Schmitt, Stefan Raible, Jürgen Wöllenstein

**Affiliations:** 1 Laboratory for Gas Sensors, Department of Microsystems Engineering-IMTEK, University of Freiburg, Georges-Köhler-Allee 102, 79110 Freiburg, Germany; Juergen.Woellenstein@ipm.fraunhofer.de; 2 ams Sensor Solutions Germany GmbH, Gerhard-Kindler-Str. 8, 72770 Reutlingen, Germany; stefan.raible@ams.com; 3 Fraunhofer Institute for Physical Measurement Techniques IPM, Heidenhofstr. 8, 79110 Freiburg, Germany; Katrin.Schmitt@ipm.fraunhofer.de

**Keywords:** gas sensor, fluoropolymer, membrane, selectivity

## Abstract

In this paper we report on how to increase the selectivity of gas sensors by using fluoropolymer membranes. The mass transport of polar and non-polar gases through a polymer membrane matrix was studied by systematic selection of polymers with different degrees of fluorination, as well as polymers whose monomers have ether groups (-O-) in addition to fluorine groups (-F). For the study, a set of application-relevant gases including H_2_, CO, CO_2_, NO_2_, methane, ethanol, acetone, and acetaldehyde as well as various concentrations of relative humidity were used. These gases have different functional groups and polarities, yet have a similar kinetic diameter and are therefore typically difficult to separate. The concentrations of the gases were chosen according to international indicative limit values (TWA, STEL). To measure the concentration in the feed and permeate, we used tin-dioxide-based metal oxide gas sensors with palladium catalyst (SnO_2_:Pd), catalytic sensors (also SnO_2_:Pd-based) and thermal conductivity sensors. This allows a close examination of the interdependence of diffusion and physicochemical operating principle of the sensor. Our goal is to increase the selectivity of gas sensors by using inexpensive fluoropolymer membranes. The measurements showed that through membranes with low polarity, preferably non-polar gases are transported. Furthermore, the degree of crystallization influences the permeability and selectivity of a polymer membrane. Basically the polar polymers showed a higher permeability to water vapor and polar substances than non-polar polymer membranes.

## 1. Introduction

The early detection of toxic and flammable gases has very high relevance in many applications and areas in order to protect human life and prevent damages to the environment and infrastructure. To date, many different gas sensors are available for all types of gases and concentrations, adapted and optimized to their specific application. The application areas include, among others, emission monitoring, safety technology, comfort, leak detection, process monitoring and condition monitoring. Comprehensive overviews of the application areas of gas analysis as well as the theoretical background of the analysis methods can be found in [1,2,3]. In many of these applications a reliable chemical sensor with reduced accuracy is sufficient. Such chemical gas sensors are distinguished from mainly optical, sophisticated systems for gas analysis [4] by the following properties: chemical sensors are small, have integrated microelectronics, can be used in mobile applications, have low power consumption and are inexpensive to manufacture by using MEMS technology. Diverse classifications of chemical sensors can be found in literature. The most widespread classification approach is done according to the physicochemical functional principle [x]. This derives to the following sensor types: thermal, mass sensitive, electrochemical, potentiometric, amperometric, conductometric and optical sensors. Biosensors are treated independently. Depending on the application and target gas concentration, a suitable gas sensor technology must be selected. Both the underlying physical and chemical sensing mechanisms as well as disturbing effects such as cross-sensitivities and environmental conditions have to be taken into account [5,6,7,8]. However, measurements which such sensors typically show problems with drift, interfering gases, vapors or uncontrollable memory effects. Especially chemical sensors, e.g., metal oxide sensors, are prone to showing false signals due to interfering gases, humidity or temperature variations [9]. Other errors may also arise due to corrosion or poisoning. One way to increase the selectivity of gas sensors in general and to protect the sensor from corrosion and poisoning is the use of suitable filter materials.

In particular, synthetic, solid membrane materials are interesting for use as selective filters in gas sensors. They can be organic, inorganic or heterogeneous membranes. Heterogeneous membranes consist of a combination of organic and inorganic materials. Although inorganic materials have a high potential as the cut-off limit and the degree of separation can be better controlled, they are normally too expensive to be used in gas sensors. It is also very difficult to produce thin and at the same time flawless crystalline layers. Existing errors such as pinholes may result in a Knudsen or viscous diffusion, which reduces the separation efficiency of the membrane. Through advanced production methods polymeric dense membranes can be manufactured inexpensively. The separation properties of these organic membranes can be easily adjusted by surface modifications. A plurality of materials for use in industrial separation processes has already been investigated relying on the different methods of gas permeation (GP), vapor permeation (VP) and pervaporation (PV) [10,11,12,13,14,15,16]. The available information in the literature on the separation properties is already comprehensive and helpful, but can only marginally serve as guidance for selective filters applicable for gas sensors. This is because of the following reasons: the permeate is always discharged in the membrane modules, very large membrane surfaces are used, and the separation processes are usually done applying a pressure difference or elevated temperature. Furthermore, usually binary mixtures (gas/gas) or (liquid/gas) are considered and the concentrations are often many times higher than the most important indicative limit values (TWA, STEL) used in gas sensing. Our aim is to investigate the permeability of symmetrical polymeric materials to H_2_, CO, CO_2_, NO_2_, methane, ethanol, acetone, acetaldehyde and moisture. The membrane has an inner diameter of ≤6.4 mm and can therefore be directly integrated into the metal caps of TO sockets, which are mainly used to mount metal-oxide gas sensors. The mass transfer takes place at room temperature and without partial pressure. The permeate is not removed.

In a previous study including polyolefins and thermoplastic polycondensates, we can see that non-polar polyolefins are not very selective, with one exception [17]. It was assumed that non-polar polymers exhibit a higher permeability for non-polar substances. A polymer with a low density has more microvoids between the chains. This results in higher gas permeability. Yet for polyolefin membranes it was observed that, contrary to expectations, the retention capacity increases with decreasing density. In contrast to the flexible and movable methane (-CH_2_-) groups in polyolefins, the linear linkage of the aromatics or heterocycles in polycondensates via an ether (-O-), ketone (-CO-), sulfone (-SO_2_-), aryl imide, ether imide, ester (-CO-O-) or a carbonate group (-O-CO-O-) leads to a stiffening of the chain links. Because of their higher strength and stiffness, thermoplastic polycondensates should exhibit lower permeability, leading to higher selectivity. Our investigation shows that polymers with slightly polar areas are very selective towards non-polar gases. For example, the presence of ether groups in the backbone favors the permeability of nonpolar gases such as H_2_.

Polymers that originate from monomers containing fluorine (F) are named fluoropolymers. A polymer of straight or branched macromolecules is referred to as a thermoplastic. For the separation of gases with small kinetic diameters primarily thermoplastics are chosen. A distinction is made between amorphous and semi-crystalline thermoplastics. Crystallization is a very complex process, because the chains have to arrange themselves in a defined crystal structure. In a polymer, irregularities in the structure of the chains always therefore occur. For this reason, a polymer material may not fully crystallize during cooling. Therefore partially crystalline thermoplastics have amorphous and crystalline phases. The degree of crystallization influences the permeability and selectivity of a polymer membrane. With increasing degree of crystallinity (i.e., increasing density) the selectivity increases, while the permeability or solubility decreases with the increase of the crystalline domains. With a low degree of crystallization, the amorphous regions predominate. This creates more microvoids between the polymer chains and results in higher gas permeability. However, this is not generally true, since certain polymers have a specific permeability to chemically similar gases or vapors. Basically, the selectivity increases with increases in the degree of crystallization and decreasing glass transition temperature. Another important property is the polarity of the membrane. Functional groups with dipole moments are, for example, fluorine groups (-F). In case the adjacent phase and the polymer are both either non-polar or polar, this results in a substantial increase in the adsorption phase and the adjacent an associated swelling of the membrane. The diffusion coefficient often increases exponentially with the volume fraction of the adsorbed material component in the membrane (Flory-Huggins equation) [18,19,20,21,22]. Basically polar polymers show a higher permeability to water vapor and polar substances than non-polar polymer membranes. Due to the differences in the structure (linear ↔ branched), the structure (amorphous ↔ crystalline), the resulting degree of crystallization, the density (low density is synonymous with large atomic distances), the polarity (polar ↔ non-polar), the glass transition point, the crystalline melting point, the separation characteristics are significantly influenced. For example, the densities of selected fluorine membranes vary between 1.37 g/cm^3^ in polyvinyl fluoride (PVF) and 2.2 g/cm^3^ in polytetrafluoroethylene (PTFE). The water absorption, being a measure of the polarity, varies from <0.01% in FEP to <0.1% in PTFE.

Fluoropolymers have in a few cases already been investigated with regard to their gas sorption properties: Sugimoto et al. [23] found that fluoropolymer films have a higher affinity for polarizable molecules than for non-polarizable. Samuel et al. [24] successfully used fluoropolymer films on field-effect transistor (FET)-based gas sensors to reduce water adsorption on the sensing surface. A fluoropolymer-based gel membrane was used in [25] to investigate CO_2_ permeability. However, no comprehensive study on fluoropolymer membranes for gas separation can be found in literature [26,27,28].

In this study we therefore now focus on the intrinsic properties of fluoropolymers and how they influence the mobility and the transport of gases through a polymer matrix. Fluorine groups can specifically influence the polarity and thus the permeability. In case the adjacent phase and the polymer are both either non-polar or polar, this results in a substantial increase in the adsorption of the adjacent phase. By increasing the volume fraction of the adsorbed substance, the diffusion coefficient increases. As already mentioned, there is a direct correlation between the presence of ether groups and the transmission of non-polar gases. For this reason, fluoropolymers containing ether groups are also used in the study. The test gases H_2_, CO, NO_2_, methane, ethanol, acetone, acetaldehyde and H_2_O (relative humidity) were used. All these gases have a similar kinetic diameter (2.6 to 4.6 Å), but have different functional groups and polarities. The concentration of gases was chosen based on the international indicative limit values (TWA, STEL). The aim of our study is to find simple relationships to predict the permeability of GP membranes towards polar and non-polar gases. In addition to this, by using different sensor types (metal oxide (MOX), catalytic bead (CB) and thermal conductivity (TC)), the influence of oxidative or reductive gases, the membrane thickness, the active surface and the humidity will be examined.

## 2. Materials and Methods

### 2.1. Fluoropolymers

**Figure 1:**
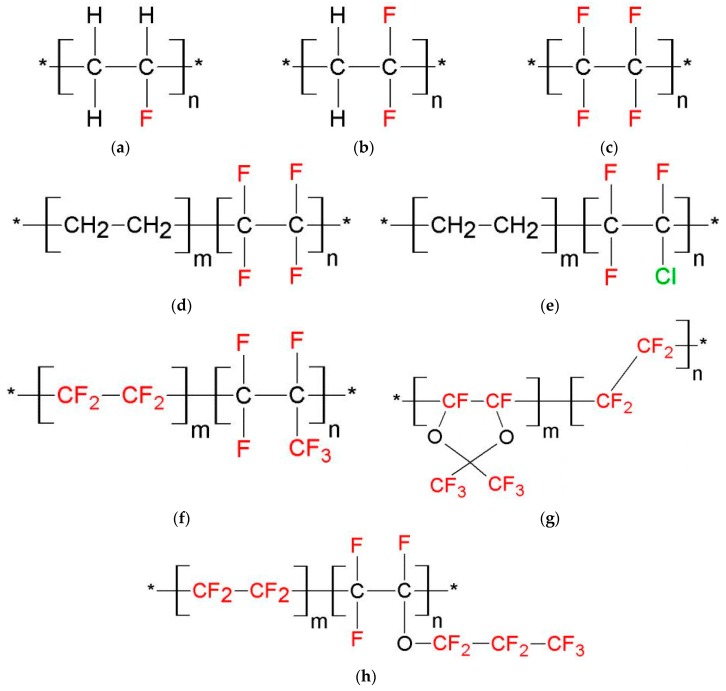
Molecular configuration of the polymers used in our study: (**a**) Polyvinyl fluoride (PVF); (**b**) Polyvinylidene fluoride (PVDF); (**c**) Polytetrafluoroethylene (PTFE); (**d**) Ethylene-tetrafluoroethylene copolymer (ETFE); (**e**) Ethylene-chlorotrifluoroethylene copolymer (ECTFE); (**f**) Tetrafluoroethylene-hexafluoropropylene copolymer (FEP); (**g**) Tetrafluoroethylene-perfluorodimethyldioxol (PDD) copolymer (Teflon^®^ AF2400); (**h**) Tetrafluoroethylene-perfluoropropylvinyl ether copolymer, perfluoroalkoxy (PFA).

The structures of the fluorine-containing polymers used are summarized in Figure 1. Fluorine-containing polymers have an almost universal chemical resistance, high thermal stabilities and low adhesivity.

The fluoropolymers were purchased from Goodfellow (GF), Reichelt Chemietechnik (RCT) and Biogeneral (BG). In the data sheets (GF) and the product catalog (RCT) we found information on the water absorption, continuous operating temperature and the density [29,30]. These were compared with the literature values on fluoropolymers [21,31,32,33,34,35]. Table 1 summarizes the physical and thermal properties of the fluoropolymers necessary for a description of the separation characteristics.

The mass transport in porous dense polymer membranes is described by the solution-diffusion model. The polymer is treated as a real liquid in which dissolved gas molecules diffuse towards a gradient. The model and its application to gas permeation in membranes are described in greater detail in [17]. In short, the mass transfer through a dense polymer membrane consists of three steps. First, the sorption of various molecules according to the distribution coefficients of the feed phase takes place. The second step is the diffusion through the membrane matrix. In the final step desorption of the gas into the permeate phase takes place. To investigate the influence not only of the type of fluoropolymer on the separation characteristics, also membranes with different thicknesses and diameters were tested.

### 2.2. MOX Sensor

A MOX sensor changes its electrical resistance upon contact with reactive gases. The operating principle of SnO_2_:Pd sensors is based on the chemisorption of gases in the presence of atmospheric oxygen. When exposed to reducing gases such as H_2_, CO, CH_4_, ethanol, acetone and acetaldehyde, the palladium oxide is reduced and excess electrons are transferred to the conduction band of SnO_2_ (n-type semiconductor). This results in an increase of the carrier concentration on the SnO_2_ surface and the resistance of the sensor is reduced. Oxidizing gases such as NO_2_ reduce the charge carrier concentration and the resistance of the sensor thus increases. The sensor signal *S* is used to describe the relation between the signal at equilibrium *x*_0_ and the signal *x* in a changing gas composition

(1) $$S=\frac{x}{x_{0}}\;\to S_{red}=\frac{R_{air}}{R_{gas}}\geq 1;S_{ox}=\frac{R_{gas}}{R_{air}}\geq 1\;.$$

*R_air_* denotes the resistance of the baseline in pure synthetic air and *R_gas_* the resistance upon exposure to the target gas. The relation of the sensor signal (*S_red_*, *S_ox_*) must be reciprocal for reducing and oxidizing gases in order to obtain positive values. The sensor signal is always ≥1. If the signal is ≅1 the resistance does not change. We now define that the sensor signal corresponds to the permeability (P).

Furthermore, the retention capacity is an important parameter to describe the separation properties of polymers. The retention capacity *R* is defined as the normalized ratio of the concentration of a solid component *i* in the permeate *p* to the concentration in the feed *f*. The sensor signals are proportional to the true concentration, which requires an adaptation of Equation (1):

(2) $${\bar{R}}_{i}=\left(1-\frac{c_{i}^{p}}{c_{i}^{f}}\right)=\underset{MOX}{\underbrace{\left(1-\frac{S_{i}^{p}-1}{S_{i}^{f}-1}\right)}};\text{ with }{\bar{R}}_{i}=\frac{1}{3}\overset{3}{\underset{i=1}{\sum }}R_{i}\;,$$

where *c* is the concentration, *S* the sensor signal of MOX sensors from Equation (1), the subscript *i* refers to a material component and the superscript p or f to the permeate and feed. SnO_2_:Pd sensors have a high reproducibility between 89 and 94, so two different sensors can be used to measure the concentration in the feed and permeate. The reproducibility (*Q*) is defined as

(3) $$Q_{x\prime }\left(p_{i}\prime \right)=\left({\frac{\frac{1}{n}\sum_{k=1}^{n}x_{k\prime }}{x\prime_{max}}|}^{p_{i}}\right)\cdot 100,$$

where

*n*: number of sensors
*x_k_'*: signal of sensor *k*
*x_max_'*: signal maximum of all sensors

In the case of *Q* = 0, the sensors are mutually not reproducible, in the case of *Q* ≈ 100 the signals are identical [36,37]. If now the retention capacity is 1, the membrane is completely impermeable for a material component. If the retention capacity is 0, then a material component can diffuse freely through the membrane. We used the metal oxide sensor AS-MLK (SnO_2_:Pd) of the company ams Sensor Solutions Germany. During the experiments the sensor was operated with a constant power of 38 mW (310 °C). The resistance to be measured is logged with the 40 Channel Single-Ended Multiplexer Module 34908A for the Data Acquisition Unit 34970A of the Company Keysight Technologies.

### 2.3. Catalytic Bead Sensor (CB)

Catalytic sensors are, strictly speaking, a mixture of a chemical and a physical sensor. The operating principle is based on a change in temperature caused by the exothermic or endothermic chemical reactions of gases with atmospheric oxygen at a catalyst and/or physically through a change in the thermal conductivity of a gas mixture. The temperature change is converted via a resistance thermometer in the form of a platinum coil (pellistor) or a planar platinum structure (e.g., on a micro hotplate) to an electrical signal and is dependent on the gas composition and the catalyst used. Usually transition metals of the platinoids partially filled d-bands (for example, Rh, Pd, Pt) are used as catalysts. This allows an exchange of electrons between the metal surface and adsorbing gases such as H_2_, O_2_ or hydrocarbons. In this chemisorption the gas particles are (usually covalently) bound to the surface of the adsorbent, whereby the activation energy is lowered [38,39]. A SnO_2_ substrate coated with Pd is used as sensitive layer. The resistance *R*_0_ of the catalytic sensor is ≈79.4 Ω. The sensor is operated using a simple voltage divider with a constant voltage. Upon gas exposure, the sensor signal then corresponds to the partial voltage *U_air_* or *U_gas_*. Through the sensor signal *S* a relation between the signal at equilibrium *x*_0_ and the signal upon gas exposure *x* can be found. Thus for the catalytic sensor the following applies:

(4) $$S=x_{0}-x\;\to S_{endo}=U_{air}-U_{gas}\;or\;S_{exo}=U_{gas}-U_{air}\;.$$

Here *U_air_* denotes the voltage of the sensor in pure synthetic air and *U_gas_* the voltage upon gas exposure. In an endothermic reaction, the resistance of the platinum structure decreases, leading to a lower voltage of the sensor. As catalytic bead sensor we used the AS-MLK (SnO_2_:Pd) of the company ams Sensor Solutions Germany. The sensor was operated at a temperature of 310 °C. The voltage to be measured is logged with the 40 Channel Single-Ended Multiplexer Module 34908A for the Data Acquisition Unit 34970A of the Company Keysight Technologies.

### 2.4. Thermal Conductivity Sensor (TC)

The principle of operation of a thermal conductivity sensor is based on the continuous measurement of the thermal conductivity difference of the sample gas stream relative to a reference gas stream. The temperature gradient is proportional to the thermal conductivity *λ* of a gas and thus specific. The signal depends on the gas concentration and Δ*λ*. Usually Pt-based structures are used as thermal conductivity sensors. The change of the thermal conductivity Δ*λ* is proportional to the change in resistance Δ*R* of the Pt structure, and thus to the voltage Δ*U*. The voltage change Δ*U* of an electrically heated Pt structure can be generally expressed as:

(5) ΔU=m · ΔR, 

where *m* is a constant. The change in the resistance of the platinum structure is given by:

(6) $$\Delta R=\left(\frac{dR}{dT}\right)\cdot \;\Delta T\;.$$

The temperature change ∆*T* is given by:

(7) $$\Delta T=\frac{\Delta P}{\left(\frac{dP}{dT}\right)}\;.$$

The temperature coefficient *dP*/*dT* is the average power dissipation based on a nominal value. The change in the power ∆*P* is given by:

(8) $$\Delta P=m^{\prime }\cdot \;\Delta \lambda_{m}.$$

By combining Equation (8) with Equation (5) the relationship between the voltage ∆*U* and a changing thermal conductivity Δ*λ* in a gas mixture is obtained:

(9) $$\Delta U=m^{\prime \prime }\cdot \;\Delta \lambda_{m}.$$

*m″* combines additional constants. In case ∆*U* is determined via a voltage divider, *m″* includes the constant total voltage *U*_0_ and the series resistor *R*_2_, in the case a Wheatstone bridge is used, *m″* includes the bridge voltage *U_Br_* and resistors *R*_2_, *R*_3_, *R*_4_ and furthermore material constants, the current I_2_, the length L of the Pt structure, the temperature difference between the filament and the ambient (*T*_f_ − *T*_a_) and an appropriate term to describe certain radii [38].

We used the thermal conductivity sensor MTCS-D1 of the company ams Sensor Solutions Germany, which was operated during the experiments with a series resistor at a constant voltage. The signal (*U_gas_* or *U_air_*) is measured as voltage drop at the voltage divider. The heater of the thermal conductivity sensor MTCS-2201 from the company Neroxis, Switzerland is pulsed at a frequency of 1 Hz and an amplitude of 0.5 V. A measuring current of a few µA was applied at the resistance thermometer. The resistance of the thermometer is determined by the temperature of the heater and the thermal conductivity of the surrounding gases. The voltage of the thermometer is filtered through two band-pass filters at a high and a low frequency. By polynomial interpolation of the two output signals and coefficients from a calibration, the gas concentration is obtained. The influence of moisture is thereby compensated [40].

### 2.5. Experimental Setup

**Figure 2:**
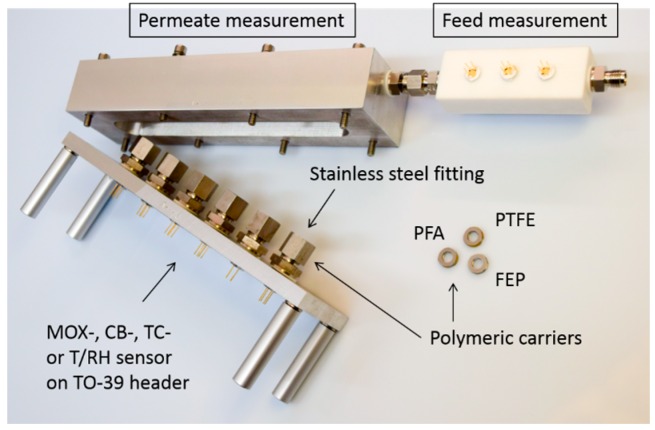
Experimental setup for study the separation properties of fluoropolymers. In the right PTFE chamber, six sensors on TO-39 header are integrated. Different types of sensors can be used to measure the gas concentration, temperature and humidity in the Feed. In the left chamber, the sensors are soldered into interchangeable stainless steel fittings to measure the gas concentration, humidity and temperature in the Permeate. The Polymeric carriers are screwed-in with EPDM and PTFE seals.

Typically, gas sensors like those used in our study are mounted on TO-39 sockets. The protective metal or plastic caps that are glued onto the sensors usually have an outer diameter of about 8 mm. For easy integration the diameter of the membrane must therefore not exceed 6.4 mm. The fluoropolymer membranes used come without support layer and have thicknesses ranging from 4.5 μm (PVDF) to 38.1 μm (Teflon AF2400). In order to prevent damage or deformation of the thin membranes during installation, they are fixed on circular stainless steel supports and screwed-in fittings as shown in Figure 2. A detailed description of the mounting procedure has been published before [4].

The experimental setup consists of two chambers connected in series, interconnected by means of Swagelok^®^. Chamber A is made of Teflon^®^ (PTFE). The chamber is used to measure the concentration, humidity and temperature in the feed. The chamber can be equipped with up to six sensors mounted on TO-39 sockets. In addition to the sensors under test, a digital temperature and humidity sensor (HYT221) from IST AG, Switzerland, was included. Chamber B is used to measure the concentration of the Permeate. The tightness of the setup was tested before the experiments were performed, as well as the sensors without membrane in both chambers. Without using a membrane, the sensor signals of the sensors in the two chambers were nearly identical. A detailed description of the experimental setup and the initial tests are also provided in [17].

Since in our gas mixing system six channels are available, two measurement profiles were generated to study the separation characteristics. In the first measurement profile H_2_ (150 ppm), CO (30 ppm), CH_4_ (1000 ppm), ethanol (500 ppm), acetone (500 ppm) and acetaldehyde (25 ppm) were measured. The concentrations of gases are based on the indicative limit values TWA. For those membranes that are permeable to at least one non-polar gas (H_2_, CO, CH_4_), a second measurement is performed with CO and CH_4_ at elevated concentrations of 100 and 3.000 ppm, as well as with the non-polar gas NO_2_ (1 ppm). The concentrations used for CO and NO_2_ are based on the STEL indicative limit values. These are given in [41,42]. The gas flow was set to 200 sccm in all measurements. As the baseline for the measurements synthetic air is used at a relative humidity of 50%.

## 3. Results and Discussion

### 3.1. Separation Characteristics of Fluoropolymers

**Figure 3:**
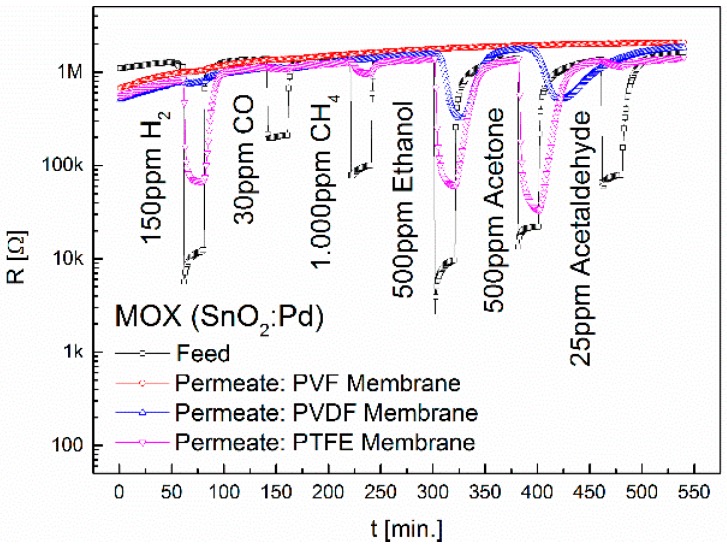
Separation properties of PVF, PVDF and PTFE upon exposure to 150 ppm H_2_, 30 ppm CO, 1000 ppm CH_4_, 500 ppm ethanol, 500 ppm acetone and 25 ppm acetaldehyde. The diameter of the membranes is 6.4 mm. The permeability of the membranes increases with the degree of fluorination.

The feed and permeate signals of the MOX sensor are plotted in Figure 3. In PVF no permeate signals can be seen when subjected to 150 ppm H_2_, 30 ppm CO, CH_4_, 1000 ppm, 500 ppm ethanol, 500 ppm acetone and 25 ppm acetaldehyde. The less polar PVDF membrane shows a high retention capacity for the gases H_2_, CO, CH_4_ and acetaldehyde. For ethanol and acetone signals of 5.5 and 4 can be obtained. These maxima take 5 min (ethanol) and 12 min (acetone) to reach after exposure to the gas. The duration of the gas exposure was 20 min. After 18 min, the sensor signal for ethanol is with a value of 4.2 already close to the maximum, while the sensor signal for acetone with a value of 1.1 is still very low. The membrane shows an increased selectivity to ethanol. The non-polar PTFE membrane shows very high retention capacities for CO, CH_4_ and acetaldehyde. For H_2_, ethanol and acetone sensor signals of 13.8, 24.8 and 44.8 were obtained, respectively. The retention capacity towards acetone (0.265) is much lower than that for H_2_ and ethanol. Unlike in PVF and PVDF, the macromolecules in the fully fluorinated PTFE do not organize in zigzag but helically. Thus PTFE has the highest density of all investigated fluoropolymers. For the PTFE membrane we observed an increased selectivity towards acetone. The permeate concentrations are below the detection limit of the TC sensor. The CB sensor shows signals for ethanol and acetone only when using the PTFE. The sensor signals for the permeates and the retention capacities are summarized in Table 2 and Table 3. Each measurement was repeated at least two times. The signal strength remain in all tested gases.

**Figure 4:**
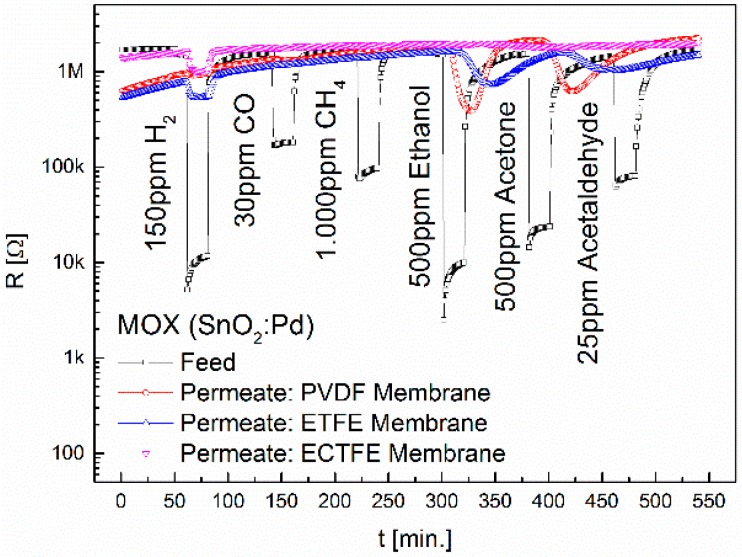
Separation properties of PVDF, ETFE and ECTFE upon exposure to 150 ppm H_2_, 30 ppm CO, 1000 ppm CH_4_, 500 ppm ethanol, 500 ppm acetone and 25 ppm acetaldehyde. The diameter of the membranes is 6.4 mm.

The feed and permeate signals of the MOX sensor are plotted in Figure 4. When comparing the permeate signals of the PVDF membrane with those of ETFE the following becomes clear: the larger CF_2_ distances caused by ethylene monomers in ETFE reduce the ethanol and acetone signals. The carbon chain in ETFE is less shielded than in PTFE and PVDF. Due to this, intermolecular forces are larger. The maxima of the sensor signals are reached even later. Additionally, we see a signal upon exposure to H_2_. The chlorination in ECTFE leads to a complete disappearance of ethanol and acetone signals. We suppose this is due to the fact that in ECTFE one fluorine atom is replaced by a chlorine atom with a lower electronegativity, so a weak dipole develops. The ECTFE membrane is impermeable to CO, CH_4_, ethanol, acetone and acetaldehyde, and thus highly selective to hydrogen.

**Figure 5:**
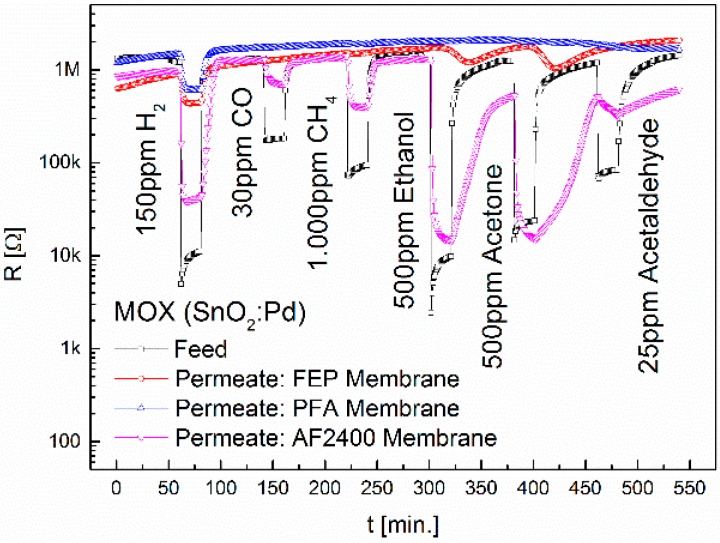
Separation properties of FEP, PFA and Teflon^®^ AF2400 upon exposure to 150 ppm H_2_, 30 ppm CO, 1000 ppm CH_4_, 500 ppm ethanol, 500 ppm acetone and 25 ppm acetaldehyde. The diameter of the membranes is 6.4 mm.

Furthermore, the influence of the introduction of side chains and heterocycles on the transport properties in fluorine membranes was investigated. For this, the fully fluorinated copolymers FEP, PFA and Teflon^®^ AF2400 were used. The results on the permeability of these membranes using the MOX sensor are shown in Figure 5.

Compared to PTFE, the trifluoromethyl substituents in FEP lead to a significant weakening of the H_2_, ethanol and acetone signals. This is against our expectations, since the -CF_3_ side chain increases the distances between neighboring macromolecules. For methane and acetaldehyde no signal is visible. The maximum in the signal for ethanol and acetone is not reached until 18 min and 21 min, respectively. This effect has already been observed in the PVDF and ETFE membranes. The propoxy group in PFA causes, in contrast to FEP, a strong retention to ethanol and acetone. The propoxy side chain (-O-R) is more flexible than the -CF_3_ side chain in FEP. Water absorption, density, degree of crystallization and melting point are almost identical. The PFA membrane as well as the ECTFE membrane is impermeable for CO, CH_4_, ethanol, acetone and acetaldehyde and therefore highly selective to H_2_, yet the permeability for H_2_ is in the PFA membrane approximately twice as high. The amorphous Teflon AF2400 membrane is permeable for all gases. The free volume is very high due to the low density. Due to the high permeability of this membrane for H_2_, ethanol and acetone also the CB and TC (MTCS2201) sensors obtain sufficiently high feed concentrations. The comparison of the highly crystalline PTFE membrane with the amorphous AF2400 membrane shows that the degree of crystallization and hence the density are suitable to only a limited extent for predicting gas permeation properties.

The permeate signals of the examined fluoropolymers are summarized in Table 2. The signals for the membranes PVDF, ETFE and FEP still increase after exposure to the target gas. The maxima are marked in the table with an asterisk. The membranes ETFE and FEP do not show a sensor signal in the permeate upon ethanol and acetone exposure.

In addition to the permeability, the retention capacity is another important parameter to describe the separation properties of polymers. The retention *R* is defined as normalized ratio of the concentration of a material component *i* in the permeate *p* to the concentration in the feed *f*. The sensor signals are simply proportional to the concentration. The sensor signals can be described by the following relationship:

(10) $${\bar{R}}_{i}=\left(1-\frac{c_{i}^{p}}{c_{i}^{f}}\right)=\underset{MOX}{\underbrace{\left(1-\frac{S_{i}^{p}-1}{S_{i}^{f}-1}\right)=\;}}\underset{CB,\;TC\left(MTCS\right)}{\underbrace{\left(1-\frac{\Delta S_{i}^{p}}{\Delta S_{i}^{f}}\right)=\;}};\text{ with }{\bar{R}}_{i}=\frac{1}{3}\overset{3}{\underset{i=1}{\sum }}R_{i}\;,$$

where *c* is the concentration, *S* the sensor signal of the MOX sensors, ∆*S* the sensor signal of the CB sensors or TC (MTCS) sensors, the subscript *i* refers to a material component and the superscript *p* or *f* to permeate and feed. Since the gas sensors have a high reproducibility, two different sensors can be used for measuring the concentration in the feed and permeate. The retention capacities of the investigated fluoropolymers are summarized in the following Table 3. The specified values are the arithmetic mean of three pairs of sensors.

If the retention capacity is 1, the membrane is impermeable for the gas. If the retention capacity is 0, then the gas can diffuse freely through the membrane. In the feed of the membranes made from ETFE, ECTFE and FEP only very low concentrations could be observed. This requires the specification of three significant digits. The standard deviation (1σ) of the membranes ETFE, ECTFE and FEP when using three pairs of sensors during the application of 150 ppm H_2_ are ±0.0004, ±0.001 and ±0.001, respectively. In a 10-fold repetition of the gas exposure to 150 ppm H_2_ to the ECTFE and PFA membranes (highly selective to H_2_), the retention capacities and standard deviations listed in Table 4 were obtained for the sensor pairs A, B and C. The uncertainty is, after 10 measurements, still in the third decimal place for each sensor pair.

**Figure 6:**
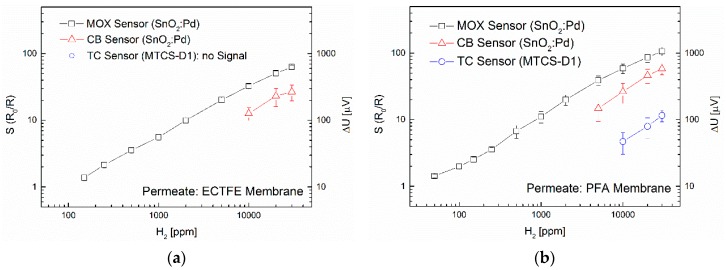
(**a**) Sensor signals upon exposure of the ECTFE membrane to H_2_ (concentration range from 150 ppm to 30,000 ppm); (**b**) Sensor signals upon exposure of the PFA membrane to H_2_ (concentration range from 50 ppm to 30,000 ppm).

To determine the limit of detection (LOD) and the linearity, an additional concentration series was measured using the hydrogen-selective membrane ECTFE and PFA. For this purpose, concentrations of 1, 3, 5, 10, 50, 100, 150, 250, 500, 1000, 2000, 5000, 10,000, 20,000 and 30,000 ppm H_2_ were applied for 20 min each. In Figure 6, the average of the three sensor types as well as their standard deviation is shown. Using the ECTFE membrane, concentrations can be detected from 150 ppm H_2_ with the MOX sensors. When using CB sensors, the LOD is significantly higher with 1 vol%. No feed signal can be observed in the TC sensors, even for 3 vol%. Using the PFA membrane, concentrations can be detected already from 50 ppm H_2_ with the MOX sensors. The LOD of the CB sensors is 5000 ppm. With the TC sensors, concentrations of at least 1 vol% (MTCS-D1) or 2.000 ppm (MTCS-2201, see Figure 9b) can be detected.

**Figure 7:**
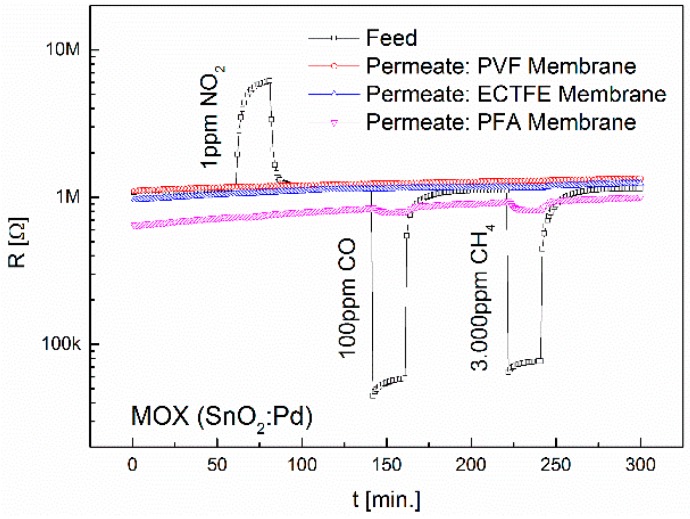
Separation properties of PVF, ECTFE and PFA upon exposure to 100 ppm CO, 3000 ppm CH_4_ and 1 ppm NO_2_. The diameter of the membranes is 6.4 mm.

The LOD of the MOX sensors is about forty times better than that of the TC sensors. In the following section we investigate the advantages of using passive filter membranes in combination with MOX sensors in greater detail. Membranes showing increased selectivity towards non-polar gases were tested again with the gases CO and CH_4_ at elevated concentrations of 100 ppm and 3000 ppm, and with the non-polar gases NO_2_ (1 ppm) and CO_2_ (1 vol%, but only with the CB and TC sensor). The concentrations for CO, CO_2_ and NO_2_ are based on the STEL guidelines. The H_2_-selective membranes ECTFE and PFA were used and additionally the PVF membrane which did not show any permeability to the gases used in the previous measurement. Assuming that an increase in gas concentration leads to potentially higher feed signals, these membranes could be further modified, e.g., through a reduction of the layer thickness or an increase in the membrane diameter. The thin layers could then be stabilized by an asymmetrical structure, by which the permeability of a membrane is only slightly affected. The results of the measurement with elevated concentrations using the MOX sensors are depicted in Figure 7.

The exposure to 1 ppm NO_2_, 100 ppm CO and 3000 ppm CH_4_ did not lead to sensor signals using the PVF and ECTFE membranes. Using the PFA membrane, sensor signals of 1.1 were obtained for CO and CH_4_. These gases can be easily distinguished by temperature modulation of the MOX sensors. In the absence of H_2_, PFA thus represents a highly selective membrane for CO and CH_4_. No signals were obtained with the CB and TC sensors because the concentrations were still below their LOD (cf. Figure 6).

### 3.2. Interdependence of Diffusion and Sensor Principle

**Figure 8:**
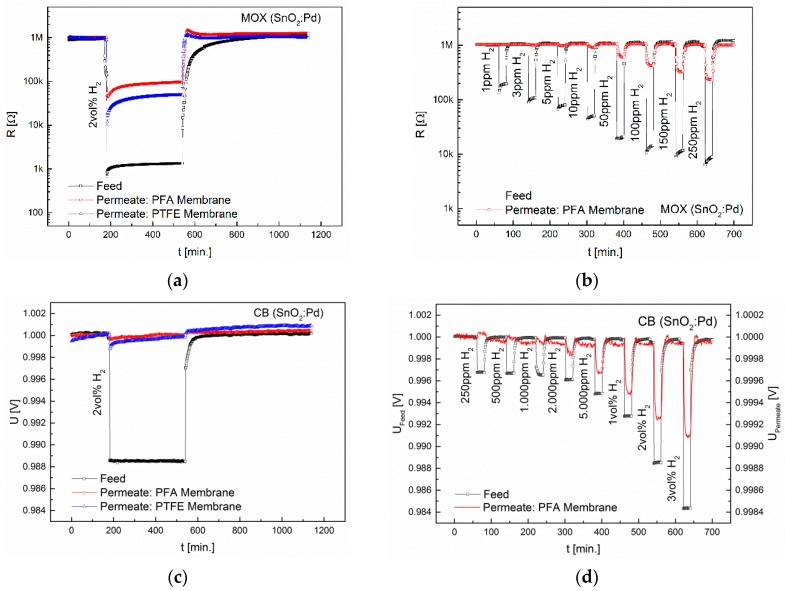
Interdependence of diffusion and sensor signal, measured with the MOX sensor. (**a**) Sensorgram of the MOX sensor in the Permeate and feed during 6 h exposure to 2 vol% H_2_ using PFA and PTFE membranes; (**b**) Sensorgram of the MOX sensor during exposure to 1–250 ppm H_2_ using the PFA membrane; (**c**) Sensorgram of the CB sensor in the Permeate and feed during 6 h exposure to 2 vol% H_2_ using PFA and PTFE membranes; (**d**) Sensorgram of the CB sensor during exposure to 250ppm-3 vol% H_2_ using the PFA membrane.

To examine the interdependence of diffusion and sensor principle, only the H_2_-selective membranes PFA and (the less selective) PTFE were chosen. Using the MOX sensors, 2 vol% H_2_ was applied for a period of 6 h. The results are depicted in Figure 8.

**Figure 9:**
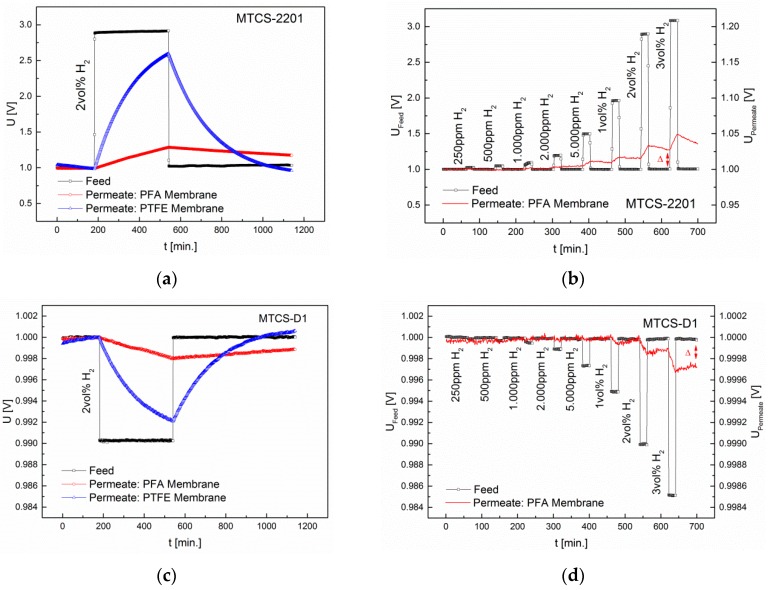
Interdependence of diffusion and sensor signal, measured with the TC sensors. (**a**) Sensorgram of the MTCS-2201 sensor (pulsed operation) in the permeate and feed during 6 h exposure to 2 vol% H_2_ using PFA and PTFE membranes; (**b**) Sensorgram of the MTCS-2201 sensor (pulsed operation) during exposure to 1–250 ppm H_2_ using the PFA membrane; (**c**) Sensorgram of the MTCS-D1 sensor (isothermal operation) in the permeate and feed during 6 h exposure to 2 vol% H_2_ using PFA and PTFE membranes; (**d**) Sensorgram of the MTCS-D1 sensor (isothermal operation) during exposure to 1–250 ppm H_2_ using the PFA membrane.

The sensor signal in the permeate shows that a diffusion equilibrium for both the PFA and the PTFE membrane is reached after ≈1 min. The equilibrium is reached when the H_2_ concentration that is desorbed in the permeate phase is equal to the concentration that is oxidized at the surface of the MOX sensor. The sensor signal remains almost constant during the exposure time. The LOD of the PFA membrane in combination with the MOX sensor is 50 ppm. The response time is in the range of 8 min. The interdependence of diffusion and TC sensors was studied with both the pulsed (MTCS-2201) and the isothermally operated (MTCS-D1) sensor. After 6 h of exposure to H_2_, a concentration gradient is still present, since it is not oxidized (as on the MOX sensor surface). The diffusion rate is primarily determined by the product of solubility and mobility. This is significantly lower in the PFA membrane. Thus, in the permeate only 15% (MTCS-2201) and 21% (MTCS-D1) of the feed concentration were reached after 6 h. The PTFE membrane has a lower selectivity and thus higher permeability or solubility. Using the PTFE membrane, in the permeate 83% (MTCS-2201) and 81% (MTCS-D1) of the feed concentration were reached after 6 h. TC sensors are indeed better suited to detect slowly increasing concentrations, on the other hand they have significantly higher response times (t_90_). Since the mass transfer from the feed to the permeate is very slow, it is therefore not possible to measure rapidly changing concentrations. This is illustrated in Figure 9.

The LOD achieved with the PFA membrane in combination with the TC sensors is 1000 ppm, which is about twentyfold higher than the LOD obtained with the MOX sensors. With the CB sensors (see Figure 8d) a LOD of 2000 ppm could be reached. The response time is identical with that obtained with the MOX sensors.

### 3.3. Influence of Membrane Thickness and Diameter

**Figure 10:**
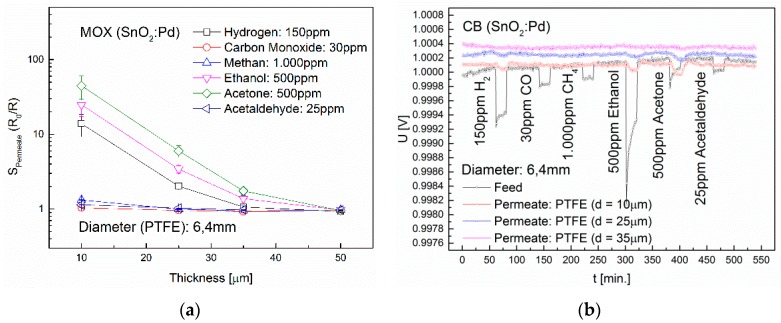
Influence of the thickness of a PTFE membrane on the diffusion of the gases H_2_, CO, CH_4_, ethanol, acetone and acetaldehyde. The membrane diameter is 6.4 mm. (**a**) Permeate signals of the MOX sensor; (**b**) Permeate signals of the CB sensor.

The permeability through a non-porous dense membrane is influenced both by the gas and the membrane properties, as well as by the thickness and diameter (i.e., area) of the membrane. In order to investigate the influence of membrane thickness on the permeability of H_2_, CO, CH_4_, ethanol, acetone and acetaldehyde, PFTE membranes were used with layer thicknesses of 10, 25, 35 and 50 µm and a diameter of 6.4 mm. The non-polar PTFE membrane shows a very high retention capacity for CO, CH_4_ and acetaldehyde. Figure 10 shows the results using MOX sensors and CB sensors. With the MOX sensors in combination with the 10 µm PTFE membrane, signals of 13.8 for H_2_, 24.8 for ethanol and 44.8 for acetone were obtained. An increase of the membrane thickness to 25 µm lead to approximately sevenfold lower sensor signals, the 35 µm thick membrane again decreased the signals roughly threefold. The results using the CB sensors are very similar, but with significantly higher LOD. With a variation in layer thickness, an increase of the selectivity is possible if the sensor signals of the material components vary greatly.

**Figure 11:**
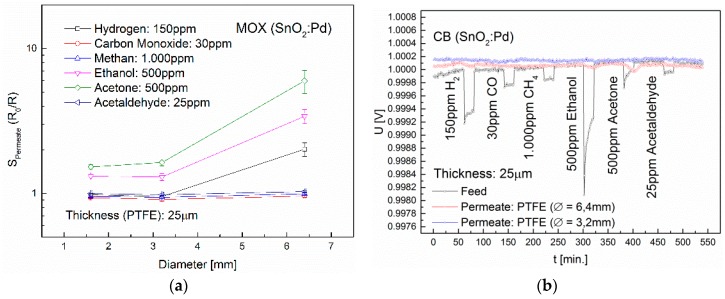
Influence of the diameter of a PTFE membrane on the diffusion of the gases H_2_, CO, CH_4_, ethanol, acetone and acetaldehyde. The membrane thickness is 25 µm. (**a**) Permeate signals of the MOX sensor; (**b**) Permeate signals of the CB sensor.

The results showing the influence of membrane diameter on the permeability of H_2_, CO, CH_4_, ethanol, acetone and acetaldehyde are depicted in Figure 11 for PFTE membranes with a diameter of 1.6 mm, 3.2 mm and 6.4 mm and a membrane thickness of 25 µm. We can see that if the membrane diameter increases from 3.2 mm to 6.4 mm, the sensor signals of the MOX sensor upon exposure to 500 ppm ethanol increases of 1.3 to 3.4 and for 500 ppm acetone from 1.6 to 6.0. Using 150 ppm H_2_, a sensor signal is only observed with a membrane diameter of 6.4 mm. Thus it is possible to minimize interferences by varying the membrane diameter.

**Figure 12:**
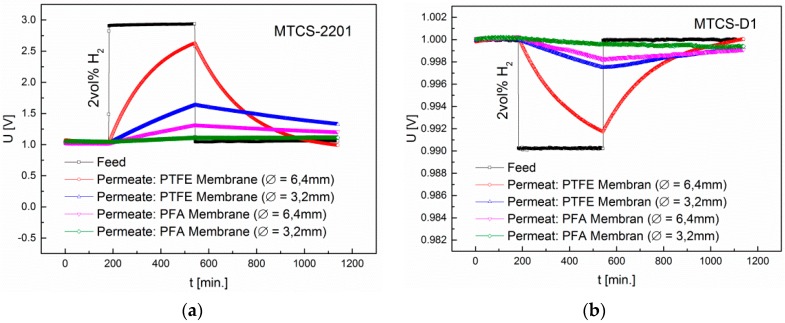
Influence of the diameter of a PTFE membrane on the diffusion of the gases H_2_, CO, CH_4_, ethanol, acetone and acetaldehyde. The membrane thickness is 25 µm. (**a**) Permeate signals of the MTCS-2201 sensor (pulsed operation); (**b**) Permeate signals of the MTCS-D1 sensor (isothermal operation).

Since for the TC sensors we did not expect signals after 20 min of gas exposure due to the low diffusion rate through the membrane matrix, membranes from PFA and PTFE with a diameter of 3.2 mm and 6.4 mm were exposed for a period of 6 h with 2 vol% H_2_. After 6 h, 33% (MTCS-2201) and 26% (MTCS-D1) of the feed concentration were reached in the permeate using the PTFE membrane with a diameter of 3.2 mm. Using a diameter of 6.4 mm, 83% (MTCS-2201) and 84% (MTCS-D1) were obtained. In contrast, only 6% (MTCS-2201) and 4% (MTCS-D1) of the feed concentration were reached in the permeate using the PFA membrane with a thickness of 3.2 mm. With an increased diameter of 6.4 mm, 16% (MTCS-2201) and 18% (MTCS-D1) were obtained (Figure 12).

When the membrane is combined with a TC sensor, a reduction of the cross-sensitivity by variation of the thickness or diameter is not possible because the gases do not chemically react.

### 3.4. Influence of Humidity on the Retention

**Figure 13:**
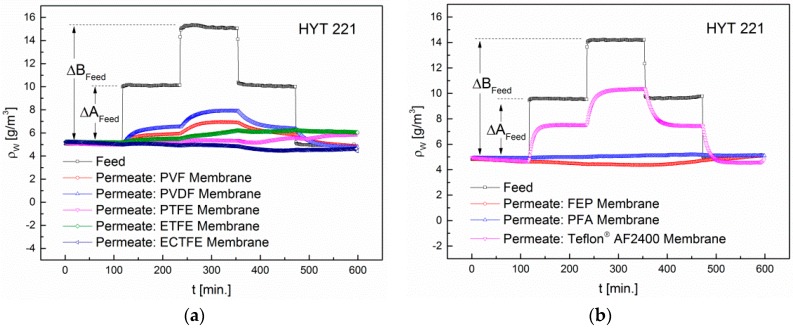
(**a**) Permeability of water vapor in PVF, PVDF, PTFE, ETFE and ECTFE membranes; (**b**) Permeability of water vapor in FEP, PFA, and Teflon^®^ AF2400 membranes. The absolute humidity was increased in two steps t of 4.8 g/m^3^ each.

Changing humidity levels are interfering signals in all three sensor types involved in our study. For this reason we also need to consider the retention of water vapor. For this, the absolute humidity was increased in two steps of 4.8 g/m^3^ each and kept constant for 2 h. The concentrations in the feed and permeate are shown in Figure 13.

The permeability to water vapor is the highest in the amorphous Teflon^®^ AF2400. Due to their high electronegativity, the chalcogen atoms in PDD lead to a strong polarization of the ring system in the 2-position. In addition, the PDD monomers increase the distance between adjacent macromolecules and lower the density to 1.74 g/cm^3^. This explains the strong increase in the absolute humidity in the Permeate to 5.7 g/m^3^. In addition to the Teflon^®^ AF2400 membrane, also the PVF, PVDF and ETFE show a permeability to water vapor, yet significantly lower. The fluoropolymers PVF and PVDF are polar, as well as water. This results in a substantial increase in the adsorption of the adjacent phase. However, the PVF membrane is not permeable to any of the gases H_2_, CO, CH_4_, ethanol, acetone, acetaldehyde and NO_2_. PVF has the lowest density of all investigated fluoropolymers (1.37–1.39 g/cm^3^). Also, the degree of crystallization is lower than in the other polymers (20%–60%). We expected that due to the lower degree of crystallinity, i.e., lower density, the selectivity is decreased, while the permeability or solubility increases compared to the other polymers. This assumption therefore is not generally valid. Table 5 shows the results for all membranes.

The copolymer ETFE consists of alternating ethylene and tetrafluoroethylene (TFE) monomers. The inductive effect described for PVDF decreases with increasing distance from the fluorine atom. Thus, the permeability to water vapor is for ETFE (1 g/m^3^) is significantly lower compared to 2.8 g/m^3^ in PVDF. The difference of ETFE and ECTFE is that in ECTFE a fluorine atom is replaced by a chlorine atom. This reduces the difference in electronegativity of 1.4 (C-F bond) to 0.6 (C-Cl bond). ECTFE is therefore polar. The densities of ETFE and ECTFE are almost identical. The permeability of water is interestingly in ECTFE, regardless of the polarity, fivefold lower than in the non-polar ETFE. In the PTFE, FEP and PFA membranes, all H atoms of the carbon backbone are replaced by fluorine atoms and the membranes are thus non-polar. This results in a high retention capacity for water vapor. The permeability of these membranes has a maximum of 0.3 g/m^3^. With the exception of PTFE, the measured permeability values for water vapor correlate very well with the literature data for water absorption.

## 4. Conclusions

In this study we focused on the intrinsic properties of fluoropolymers and how they influence the mobility and the transport of gases through a polymer matrix, since fluorine groups can specifically influence the polarity and thus the permeability. The test gases H_2_, CO, NO_2_, methane, ethanol, acetone, acetaldehyde and H_2_O (relative humidity) were used. All these gases have a similar kinetic diameter (2.6 to 4.6 Å), but have different functional groups and polarities. The aim of our study was to find simple relationships to predict the permeability of fluoropolymer membranes towards polar and non-polar gases. Using different sensor types, the influence of oxidative or reductive gases, the membrane thickness, the active surface and the humidity was examined. With the arrangement of the fluorine atoms (symmetrical ↔ asymmetrical) in fluoropolymers, it is possible to specifically vary the polarity of the membrane to separate polar from non-polar gases. For example, the polar PVDF membrane shows high selectivity to ethanol. A cross-sensitivity is observed in acetone only at prolonged exposure (≥20 min). Compared to this, the PTFE membrane has a very low retention capacity for acetone and therefore an increased selectivity. The membranes ECTFE and PFA are highly selective to H_2_. The PFA membrane had a thickness of 25 µm and a diameter of 6.4 mm. When the thickness is reduced or the surface increased, the permeability to the gases CO and CH_4_ increases. This has been demonstrated indirectly through an increase in the gas concentration. During pulsed operation of the MOX sensor, these gases can also be easily differentiated. The modification of the PFA membrane then leads to a high selectivity towards CO and CH_4_ in the absence of H_2_. Yet due to their low permeation rate, these membranes have only limited use in combination with CB or TC sensors. When using TC sensors it is not possible to reduce the cross-sensitivity by varying membrane thickness or diameter, because the gases do not chemically react on a TC sensor surface. This leads to very long response and recovery times. Our measurements showed that through membranes with low polarity (inductive effect), preferably non-polar gases are transported. The degree of crystallization influences the permeability and selectivity of a polymer membrane. With an increasing degree of crystallinity (increasing density) the selectivity increases, while the permeability/solubility decreases with an increase of the crystalline domains. Basically polar polymers show a higher permeability to water vapor and polar substances than non-polar polymer membranes. Therefore, fluoropolymers such as PCTFE and Nafion^®^, which were not included in our study, should have a high potential as a selective filter for non-polar gases. We found, with the exception of PTFE, a good agreement between literature values for water absorption and our measured permeability towards water vapor. The consideration of the surface energy of the membranes could be used in future work to better predict the permeability of polar/non-polar gases.

## Figures and Tables

**Table 1:** Selected structural, thermal and mechanical properties of the fluoropolymers used in our study. dc refers to the degree of crystallinity, the glass transition temperature is T_G_, T_M_ the crystallite melting point, T_CO_ the continuous operating temperature, WA is water absorption in 24 h based on weight percent, the density ρ and z the thickness of the membrane sheet. Adapted from data sheets of the suppliers GoodFellow (GF), Reichelt Chemietechnik (RCT) and Biogeneral (BG) [14,15].

Polymer	Structure at RT (21 °C)	T_G_ (°C)	T_M_ (°C)	T_CO_ (°C)	WA (24 h, m%)	ρ (g/cm^3^)	z (μm)
PVF	partially crystalline	−80; −20; 50; 150	200	150–200	0.05	1.37–1.39	13 (GF)
	(dc 20%–60%)						
PVDF	partially crystalline	−70; −38; 50; 100	171	135–150	0.04	1.76	4.5 (GF)
	(dc 35%–70%)						
PTFE	partially crystalline	−97; 19; 30; 127	327	260	0.1	2.2	10 (RCT)
	(dc 55%–90%)						
ETFE	partially crystalline	−120; −25; 110	270	150–160	≤0.03	1.7	25 (GF)
	(PTFE: 75%)						
ECTFE	partially crystalline	−65; 90; 140	240	130–170	<0.02	1.68	12.5 (GF)
	(dc 50%–60%)						
FEP	partially crystalline	290	290	250	<0.01	2.12–2.17	25 (RCT)
	(dc 40%–50%)						
PFA	partially crystalline	−100; −5; −30; 90	305	260	<0.03	2.15	25 (GF)
	(dc > 48%)						
Teflon^®^ AF2400	amorphous	240	-	260	-	1.74	38.1 (BG)
	(87 mol % PDD)						

**Table 2:** Permeate signals of the MOX, CB and TC sensors using PVF, PVDF, PTFE, ETFE, ECTFE, FEP, PFA and Teflon^®^ AF2400 membranes. The values given are the arithmetic mean of three measurements. For some permeate signals (*), the maximum is reached after ≥20 min.

Membrane	Sensor	H_2_	CO	CH_4_	Ethanol	Acetone	Acetaldehyde
PVF	MOX	-	-	-	-	-	-
PVDF	MOX	-	-	-	4.2 (*5.5)	1.1 (*4)	-
PTFE	MOX	13.9	-	1.3	24.8	44.8	1.1
	CB	-	-	-	68.6	137.3	-
ETFE	MOX	1.4	-	-	*2	*1.4	-
ECTFE	MOX	1.4	-	-	-	-	-
FEP	MOX	1.9	-	-	*1.3	*1.6	-
PFA	MOX	3	-	-	-	-	-
Teflon^®^ AF2400	MOX	24	1.9	3.7	104.7	29.4	1.1
	CB	664.9	-	-	1253.9	787.7	-
	TC	11.2	-	-	3.2	3.3	-

**Table 3:** Retention capacities of the PVF, PVDF, PTFE, ETFE, ECTFE, FEP, PFA and Teflon^®^ AF2400 membranes. The values given are the arithmetic mean of three measurements. For some retention capacities (*), the maximum is reached after ≥20 min.

Membrane	Sensor	H_2_	CO	CH_4_	Ethanol	Acetone	Acetal-Dehyde
PVF	MOX	-	-	-	-	-	-
PVDF	MOX	-	-	-	*0.976	*0.965	-
PTFE	MOX	0.862	-	0.975	0.827	0.265	0.990
	CB	-	-	-	0.909	0.194	-
ETFE	MOX	0.997	-	-	*0.994	*0.994	-
ECTFE	MOX	0.997	-	-	-	-	-
FEP	MOX	0.991	-	-	*0.998	*0.989	-
PFA	MOX	0.997	-	-	-	-	-
Teflon^®^ AF2400	MOX	0.757	0.831	0.794	0.267	0.447	0.992
	TC	0.292	-	-	0.699	0.743	-

**Table 4:** Retention capacities of the H_2_-selective ECTFE and PFA membranes. The values given are the arithmetic mean and standard deviation of ten measurements at 150 ppm H_2_.

Membrane	Sensor Pair A	Sensor Pair B	Sensor Pair C
ECTFE	0.995 ± 0.001	0.995 ± 0.001	0.995 ± 0.001
PFA	0.995 ± 0.001	0.995 ± 0.001	0.995 ± 0.001

**Table 5:** Permeability of the PVF, PVDF, PTFE, ETFE, ECTFE, FEP, PFA and Teflon^®^ AF2400 membranes to water vapor upon variation of the absolute humidity. ∆A and ∆B refer to the concentration steps shown in Figure 13. The Permeate and feed concentrations are measured using the T/RH sensor HYT221 from IST AG, Switzerland.

Membrane	∆A_feed_ (g/m³)	∆B_feed_ (g/m³)	∆A_permeate_ (g/m³)	∆B_permeate_ (g/m³)
PVF	5.1	10	0.8	1.7
PVDF	5	9.8	1.5	2.8
PTFE	4.8	9.4	0.2	0.3
ETFE	4.9	10.1	0.3	1
ECTFE	4.9	9.7	0.1	0.2
FEP	4.8	9.4	0.2	0.3
PFA	5	10.1	0.1	0.2
Teflon^®^ AF2400	4.8	9.4	2.9	5.7

## Abbreviations

ECTFE	ethylene-chlorotrifluoroethylene copolymer
ETFE	ethylene-tetrafluoroethylene copolymer
FEP	tetrafluoroethylene-hexafluoropropylene copolymer
FET	field effect transistor
LOD	limit of detection
MEMS	microelectromechanical system
MOX	metal oxide
PFA	tetrafluoroethylene-perfluoropropylvinyl ether copolymer, perfluoroalkoxy
PDD	tetrafluoroethylene-perfluorodimethyldioxol
PTFE	polytetrafluoroethylene
PVDF	polyvinylidene fluoride
PVF	polyvinyl fluoride
SCCM	Standard Cubic Centimeters per Minute
STEL	short-term exposure limit
TWA	Time-weighted average or permissible exposure limit
TC	thermal conductivity
